# Muscle wobbling mass dynamics: eigenfrequency dependencies on activity, impact strength, and ground material

**DOI:** 10.1038/s41598-023-45821-w

**Published:** 2023-11-09

**Authors:** Kasper B. Christensen, Michael Günther, Syn Schmitt, Tobias Siebert

**Affiliations:** 1 Motion and Exercise Science, University of Stuttgart, Stuttgart, Germany; 2 Computational Biophysics and Biorobotics, University of Stuttgart, Stuttgart, Germany; 3 Friedrich–Schiller–University, Jena, Germany; 4 Stuttgart Center for Simulation Science (SC SimTech), University of Stuttgart, Stuttgart, Germany

**Keywords:** Musculoskeletal models, Animal behaviour, Biomechanics

## Abstract

In legged locomotion, muscles undergo damped oscillations in response to the leg contacting the ground (an impact). How muscle oscillates varies depending on the impact situation. We used a custom-made frame in which we clamped an isolated rat muscle (*M. gastrocnemius medialis* and *lateralis*: GAS) and dropped it from three different heights and onto two different ground materials. In fully activated GAS, the dominant eigenfrequencies were 163 Hz, 265 Hz, and 399 Hz, which were signficantly higher (p < 0.05) compared to the dominant eigenfrequencies in passive GAS: 139 Hz, 215 Hz, and 286 Hz. In general, neither changing the falling height nor ground material led to any significant eigenfrequency changes in active nor passive GAS, respectively. To trace the eigenfrequency values back to GAS stiffness values, we developed a 3DoF model. The model-predicted GAS muscle eigenfrequencies matched well with the experimental values and deviated by − 3.8%, 9.0%, and 4.3% from the passive GAS eigenfrequencies and by − 1.8%, 13.3%, and − 1.5% from the active GAS eigenfrequencies. Differences between the frequencies found for active and passive muscle impact situations are dominantly due to the attachment of myosin heads to actin.

## Introduction

Muscles are soft tissues visco-elastically connected to the comparatively rigid skeletal system. Muscles can be displaced relative to the bone in response to a leg’s ground impact during terrestrial locomotion due to the soft coupling and distributed inertia within the musculoskeletal systems^1–3^. Such relative displacements of muscle masses are named “wobbling mass”^1,2,4^ dynamics. They are crucial factors in high-impact responses^4^, such as jumping or running, in which they critically shape the courses of the ground reaction force (GRF)^5,6^ as well as the joint moments and forces^2–4,7^, and significantly contribute to energy dissipation^8–11^.

The most common way to study wobbling mass dynamics in humans is either by using high-speed cameras to capture skin marker displacement following touch-down (TD)^2,7,9^ or by measuring the change in the electrical muscle activation in response to various types of impact strengths^12,13^ or ground properties^1,12,14,15^. Accordingly, Nigg and Wakeling (2001)^13^ introduced the concept of “muscle tuning”, arguing that muscles can tune (by changing activation patterns and thus the activity courses during impact responses) muscle fibre stress and thus stiffness and damping to avoid possible resonance. Resonance may occur if the frequencies of the input signal (deceleration of the bone) and the muscle responding by its wobbling-mass properties, i.e. its eigenfrequencies, are similar^13,16^.

How the muscle is tuned in impact situations depends, amongst other things, on muscle activation level^13^ and the interaction of mechanical lower limb and ground properties^15^. However, a disadvantage of examining human subjects to determine muscle frequencies in vivo is the potential mechanical effect of neighbouring bones or soft-tissue structures, such as fat and adjacent muscles, on the target muscle. In some cases, the momentum of attached devices, like accelerometers, let alone the potential systematic measurement errors due to skin attachment, which likely amplifies due to any device’s inertia and the skin movement artefacts (in determining *bone* positions), with the latter potentially also occurring even for markings painted on the skin^2^. In addition, there are regionally varying contributions to the overall whole-muscle oscillation^17^ because of the complex interaction between properties of tendons^18,19^, aponeurosis^20,21^, and muscle fibre material^17^.

So far, little is known of how the oscillation frequencies of muscles depend on the impact strength, as it is hard to address this issue with in vivo human experiments. Other than some activity dependence^1,14^, it has been suggested that the oscillation frequencies of muscles after an impact, or at least the dominant frequency, are independent of impact strength^22^. This is plausible because the stiffness of the fibre material likely stays the same if there is no change in muscle activity, i.e. the number of formed cross-bridges (CB) remains the same. However, an increase in impact strength leads to an increased oscillation amplitude^22^. Thus, depending on the impact strength, the CBs’ displacement paths on their force-length and force-velocity curve change, thereby potentially even changing these characteristics in themselves, which would shift the muscle’s oscillation frequencies.

Previously^17^, we have developed an experimental setup to analyse the wobbling mass dynamics of an isolated rat GAS muscle. For emulating a leg impact in rat locomotion, we dropped specimens of isolated GAS, clamped into a C-shaped frame^11,17^, onto a polystyrene cushion. Furthermore, we estimated the main eigenfrequency of a fresh and fully active GAS to be about 210 Hz. A huge advantage of our ex vivo setup is the direct control of the GAS’ initial impact conditions, i.e. its (isometric) force, the spatial position, the impact strength (e.g. by dropping height), and the deceleration time (e.g. by the frames ground contact material properties). Thus, GAS oscillatory responses can be manipulated by independently varying these conditions and properties. By making full use of our ex-vivo setup, this paper aims to determine the eigenfrequencies of fully activated and passive (GAS) muscle for different impact strengths (dropping heights) and ground material properties (polystyrene and aluminium). Secondly, we aim to trace several eigenfrequency values back to stiffness values of the main constituents, the fibre material, the tendons, and the aponeuroses’ regions.

## Methods

## Ethics

We performed all experiments on muscles (*M. gastrocnemius medialis* and *lateralis*: GAS) of freshly killed rats (*Rattus norvegicus*, Wistar). All experiments were carried out in accordance with ARRIVE guidelines and recommendations of the German animal welfare law (Tierschutzgesetz, §4 (3)). The protocol of this study was approved by the competent authority for animal welfare in Baden-Württemberg, Germany (Regierungspräsidium Stuttgart, Permit Number: 35-9185.81/0491). We anaesthetised the rats with sodium pentobarbital (100 mg per 1 kg body mass).

## Experimental setup

**Figure 1:**
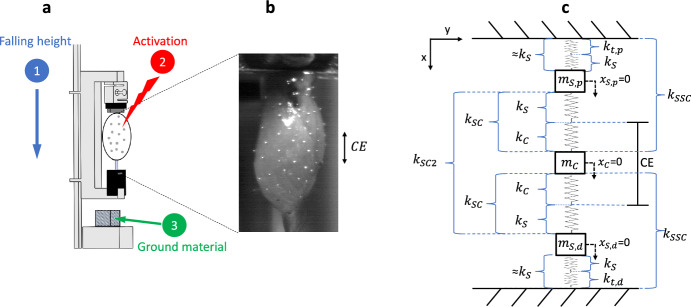
The experimental used frame with an inserted GAS and the developed GAS model with 3 degrees of freedom (3DoF). (**a**) shows the experimental setup with the three conditions varied: changing the impact strength by dropping the aluminium frame from different heights (blue, 1), changing the muscle activation, i.e. GAS being either passive or active (red, 2) and changing the ground material from polystyrene to aluminium (green, 3). (**b**) is an enlarged captured frame of GAS with CE being the length of the region that contains only fibre material. (**c**) is the fixated GAS described as an undamped system modelled by three masses connected by four compound springs. Here, the most proximal and the most distal of the springs are equally stiff ($k_{S}$) and assumed to consist of tendon aponeurosis, and fibre material properties, with tendons ($k_{t,p|d}$) being generally at least an order of magnitude stiffer than the other elements, thus, almost non-relevant contributors to combined stiffnesses $k_{S}$ and $k_{SC}$. For simplification, our second-order muscle model is thus assumed to be completely symmetrical, equipped with only two stiffnesses $k_{S}$ and $k_{SC}$, which implies $k_{SSC}=k_{S}+k_{SC}$ and $k_{SC2}=k_{SC}+k_{SC}$ [See Eqs. (4) and (5)]. The two compound springs ($k_{SC}$), which are attached to the mass representing sole fibre material in the GAS centre ($m_{C}$), consist of a $k_{S}$ in series with the contractile element stiffness ($k_{C}$). Each assumed mass of a region (portions of GAS muscle mass: $m_{S,p}=m_{S,d}=m_{S}$) is located between two identical $k_S$ springs. $x_{S,p}=0$, $x_{C}=0$, and $x_{S,d}=0$ are the initial positions of $m_{S,p}$, $m_{C}$, and $m_{S,d}$ in the *x*-direction, respectively.

Each GAS was dissected from its surrounding tissues, fixated (clamped) in a custom-made aluminium frame, and dropped from a pre-determined height on either polystyrene or aluminium (Fig. 1a). To measure muscle wobbling mass dynamics, the frontal surface of the muscle belly was patterned stochastically with high-grade steel markers (spheres, nominal diameter 0.4 mm, mensuration N0, IHSD-Klarmann, 96047 Bamberg, Germany) (Fig. 1b). These steel markers were held in place by the adhesive surface of the muscle belly in the same manner as the blunt bent wire that extended from the lower clamp (same procedure as in^11,17^). Before and after touch-down (TD) of the frame, we captured local muscle kinematics with two high-speed cameras (HCC-1000 BGE, VDS Vosskühler, 07646, Germany), each of which recorded with 256 × 1024 pixels per sample at 1825 Hz sampling rate. The frame consisted of a backbone with two extrusions to form an almost square C-shape, all made in aluminium.

The aluminium frame had mounted beneath the tip of the upper extrusion, in that order (all screwed to each other), a force transducer, an insulator, and the upper clamp for muscle fixation (Fig. 1a). Above the lower extrusion tip, an insulator was mounted to it, followed by the lower clamp for muscle fixation. Thus, the muscle was electrically insulated from the rest of the setup. We applied direct muscle stimulation (Aurora Scientific 701C) by 500 µs long square wave pulses of 12 V (three times the twitch threshold) generated at 100 Hz to ensure tetanic contraction^23^. We defined the optimal fibre length ($L_{opt}$) as the GAS length measured with the knee and ankle joints at 90° ($L_{GAS,90^{\circ }}$), plus an added 2 mm ($L_{GAS,90^{\circ }}\,+\,2\,mm{\,=\,}L_{opt}$), with the latter value inferred from literature^23,24^. This experimental setup is described in more detail elsewhere^11^.

## Experimental trials

In this study, we used our GAS specimens (N = 25) to conduct the following trials: dropping GAS onto polystyrene from either 1 cm, 1.5 cm, or 4 cm height, as well as dropping GAS onto aluminium from 2 mm. We labelled these trials group 1 (1 cm trials), group 2 (1.5 cm trials), group 3 (4 cm trials), and group 4 (aluminium trials), respectively, which contained both active (A$_i$) and passive (P$_i$) trials within each group 1–4 (Table 1). All trials within each group started with fully stimulated GAS trials, ensuring maximal (initially non-fatigued) isometric force ($F_{max}$) at TD, followed by trials without muscle stimulation, i.e. passive GAS trials.

**Table 1:** Anatomical data given as the mean value ± standard deviation.

Description	Unit	Group 1	Group 2	Group 3	Group 4
		1 cm	1.5 cm	4 cm	Alu (2 mm)
Animal mass	g	408.4 ± 16.7	518.1 ± 11.3	417.5 ± 15.0	454.4 ± 38.0
GAS mass ($m_{GAS}$)	g	1.9 ± 0.1	2.5 ± 0.1	2.1 ± 0.2	2.4 ± 0.3
GAS length at $90^{\circ }$ ($L_{GAS,90}$)	mm	43.7 ± 1.2	46.2 ± 0.7	42.8 ± 0.5	45.0 ± 0.9
GAS length in frame$^{\dag }$	mm	44	48	45	47
Belly length ($L_{belly}$)	mm	32	35	33	34
Proximal tendon length$^{\dag }$	mm	2	2	2	2
Distal tendon length	mm	10.4 ± 0.6	10.5 ± 0.3	10.0 ± 0.4	10.6 ± 0.5
Total tendon length	mm	12	13	12	13
ACSA ($A_{CE,max}$)	mm^2^	96.4 ± 6.1	96.5 ± 3.7	101.0 ± 4.6	96.0 ± 2.9
	Average*				
GAS mass ($\bar{m}_{GAS}$)	g	2.2 ± 0.3	–	–	–
ACSA ($\bar{A}_{CE,max}$)	mm$^2$	97.5 ± 2.6	–	–	–
average belly length ($\bar{L}_{belly}$)	mm	33.5 ± 1.3	–	–	–
distance to ACSA($\bar{L}_{CE,max,y}$)	mm	19 ± 1.4	–	–	–

We calculated the anatomical cross-sectional area (ACSA) right before TD by assuming that the belly had the geometrical shape of a half-ellipse.

$^{{\dag }}$ The 2 mm added to measured $L_{GAS,90}\approx L_{opt}$^23,24^, and the proximal tendon length of 2 mm^23^ were both inferred from the literature. group1, group2, group3 and group4 are labels for the 1 cm, 1.5 cm, 4 cm and aluminium (2 mm) trials, respectively.

*The average values for each group 1, group 2, group 3, group 4.

## Spectral analysis

We used our captured time-velocity data of GAS to determine frequency spectra. We analysed the data between TD and the instant at which the acceleration of the arithmetic mean of all belly markers ($a_{COM}$) returned to zero for the second time (after $\approx$ 17 ms). The position of the GAS’ centre of mass (COM) was estimated with the kinematic information from all markers (arithmetic mean) as in previous studies^11,17^.

To transform our time domain signal into the frequency domain

1 $$\begin{aligned} Y(k)=\sum _{n=0}^{N-1} y(n)\cdot {e}^{\frac{-2\pi \cdot i\cdot k \cdot n}{N}}\quad , \end{aligned}$$

We used the Cooley–Tukey algorithm^25,26^ [Eq. (1)], with *N* being the number of samples, *n* the time index, *k* the frequency index, *y*(*n*) the input signal amplitude at sample *n*, and *i* the imaginary unit defined by $i =: \sqrt{-1}$. Due to the short length of the analysed period, the frequency resolution of our captured time-velocity data would have actually been only about 59 Hz (1$\div$0.017 s). However, the captured time domain data were extended by padding trailing zeros to create 256-point data sets, which methodically provided a frequency resolution of about 7.1 Hz (1825 Hz$\div$256). In each trial, we normalised the frequency amplitudes of the calculated spectrum to their maximum value ($Y_{0}$).

## GAS 3DoF model: spring stiffness

To look for frequencies due to GAS oscillations in the spectral analysis, we treated the GAS as an undamped system with three point masses in series connected by linear springs (3DoF model, Fig. 1c), with the sum of the point masses being the GAS’ mass ($m_{GAS}$). This model system is fixated at both ends (clamped between origin and insertion) and can oscillate in only one dimension, namely the GAS’ longitudinal axis (Fig. 1c). To explain our model and the reasoning behind our assumptions, we have separated the GAS 3DoF model method section into three sections: “spring stiffness”, “mass”, and “eigenfrequencies and local muscle stiffnesses”.

With our simple model approach, we assume that the muscle belly and aponeuroses are similar in shape and elastic properties proximally and distally. Moreover, we separate the GAS region that solely contains fibre tissue from the rest of the GAS tissues. Accordingly, we do not consider inhomogeneities^27,28^ and anisotropies^21^ of the aponeuroses at this stage, nor the complex interaction of aponeuroses and fibre material in their overlapping zones. Such a 3DoF model would allow for differences in proximal and distal tendon stiffnesses ($k_{t,p}$ and $k_{t,d}$, respectively) due to differences in proximal and distal tendon lengths (Table 1). However, because Young’s modulus of leg tendon material is $\approx$1.5 GPa^18,19^, which makes any estimated $k_{t,p}$ or $k_{t,d}$ minimally 10 and up to 50 times higher, respectively, than each (proximal or distal) combined aponeurosis and part of fibre stiffness in fully active GAS (the stiffness of the combination of the two tendon-aponeurosis-fibre complexes termed $k_{TAC}$^17^), we simplify the elastic properties of the most proximal and distal (the ‘outer’) springs (Fig. 1c) to each the same stiffness

2 $$\begin{aligned} k_S\approx \left( \frac{1}{k_{t,p}}+\frac{1}{k_{S}}\right) ^{-1} \approx \left( \frac{1}{k_{S}}+\frac{1}{k_{t,d}}\right) ^{-1}\quad . \end{aligned}$$

As a consequence, the other two (‘inner’) springs connecting the mass in the centre of the muscle model to the other two masses are likewise identical (their stiffness: $k_{SC}$, see Fig. 1c). Both ‘inner’ springs are composed of the stiffness of fibre material solely ($k_{C}$) in series with $k_{S}$:

3 $$\begin{aligned} k_{SC}=\left( \frac{1}{k_{S}}+\frac{1}{k_{C}}\right) ^{-1}\quad . \end{aligned}$$

In both directions, the stiffness of the fibre material $k_C$ is arranged adjacently to $m_C$ (Fig. 1c). As we assume proximal-distal stiffness symmetry, we further say that

4 $$\begin{aligned} k_{SSC} = k_{S} + k_{SC} = k_{S} + \left( \frac{1}{k_{S}}+\frac{1}{k_{C}}\right) ^{-1}\quad \end{aligned}$$

and

5 $$\begin{aligned} k_{SC2}=2\cdot k_{SC}= \left( \frac{1}{k_{S}}+\frac{1}{k_{C}}\right) ^{-1}+\left( \frac{1}{k_{S}}+\frac{1}{k_{C}}\right) ^{-1}\quad \end{aligned}$$

as the springs on either side of a point mass ($m_{S,p}$, $m_{C}$, or $m_{S,d}$) act in parallel (see the cubic equation, Supplementary Text S1), which is also indirectly seen in Eq. (10), where the eigenvector of $m_C$ is zero (eigenvector for $freq _{2}$, Supplementary Eq. S20, p. 5).

## GAS 3DoF model: mass

As seen in Fig. 1c, the lumped mass located in the centre of our model ($m_{C}$) is attached to only $k_{C}$; hence, that mass signifies fibre material only. We estimate the mass

6 $$\begin{aligned} 0.68\,\text {g} = m_{C} = 0.75\,\text {cm}\cdot 0.86\,\text {cm}^2\cdot 1.06\,\frac{\text {g}}{\text {cm}^3}, \end{aligned}$$

located in this central, fibre-only region of GAS, with 0.75 cm and 0.86 cm^2^ being its length and average ACSA ($ACSA_{avr}$)^11^, respectively, and 1.06 g/cm^3^ the fibre material density^29^. Therefore, we can reasonably assume both stiffness and mass symmetry, namely

7 $$\begin{aligned} \frac{m_{GAS}}{3} =0.63\,\text {g} = m = m_{S,p} = m_C = m_{S,d}, \end{aligned}$$

because $m_{GAS}$ = 1.9 g (Table 1; mean group1 value of 1.9 g are the same in our present and earlier^11^ study). From Eq. (6), it is also clear that fibre material mass dominates $m_{S,p|d}$ because overall tendon mass, grossly, only makes up 4% ($\frac{0.03\,\text {g}}{0.63\,\text {g}}$):

8 $$\begin{aligned} m_{t,p} + m_{t,d} = 0.03\,\text {g} = 1.2\,\text {cm} \cdot 0.024\,\text {cm}^2 \cdot 1.12\,\frac{\text {g}}{\text {cm}^3} . \end{aligned}$$

In Eq. (8), 1.12  g/cm^3^ is the density of tendon^30^, 0.024 cm^2^ the Achilles tendon ACSA (mean value from^11,17^), and 1.2 cm the total tendon length (Table 1).

## GAS 3DoF model: eigenfrequencies and stiffnesses comparisons to literature

In this study, the experimental setup and the captured time displacement data is the same as in Christensen et al.^11^, which also included the same passive and $F_{max}$ trials as the 1 cm trials here (group 1). For this reason, we only applied our 3DoF model (Fig. 1c) on passive (P1) and active (A1) group1 trials.

We grossly assume that the muscle has at least three separable regions: each a proximal and distal tendon-aponeurosis-fibre region, which are mechanically identical [Eqs. (7) and  (2)], plus a fibre-only region in the muscle centre. Hence, in the core of our model, we have only two stiffness parameters, which are $k_S$ and $k_C$, respectively, and three masses *m* of identical value [Eq. (7)]. Because of this simplistic approach, the model is separable into units of either $k_{SSC}$ [Eq. (4)] or $k_{SC2}$ [Eq. (5)]; as such, the eigenfrequencies of the model are

9 $$\begin{aligned} freq _{1,3}&= \frac{1}{2\pi } \cdot \sqrt{\frac{1}{m} \cdot \frac{{k_{SSC}+k_{SC2} \pm \sqrt{(k_{SSC}+k_{SC2})^2 - 4\cdot k_{SC2} \cdot k_{SSC}-k_{SC2}^2 \cdot 2^{-1}}}}{2}} \end{aligned}$$

10 $$\begin{aligned} freq _{2}&= \frac{1}{2\pi }\cdot \sqrt{\frac{k_{SSC}}{m}}=\frac{1}{2\pi } \cdot \sqrt{\frac{k_S+k_{SC}}{m}} \end{aligned}\quad.$$

For more information regarding the deduction of Eqs. (9) and (10), and if $m_{S,p}=m_{S,d}\ne m_C$, see Supplementary Text S1.

In our previous study^11^, the whole GAS’ muscle-tendon-complex was modelled by just one overall muscle mass (measured) suspended on the frame by one overall spring with a stiffness $k_{MTC}$ (measured), and the latter was further assumed to consist of a serial arrangement of an inferred overall tendon-aponeurosis-complex stiffness $k_{TAC}$ and a measured fibre material stiffness $k_{CE}$ of the fibre-only (contractile element, CE) muscle centre:

11 $$\begin{aligned} k_{MTC}&=\left( \frac{1}{k_{TAC}}+\frac{1}{k_{CE}}\right) ^{-1}&\quad . \end{aligned}$$

With both $k_{MTC}$ and $k_{CE}$ [Eq. (11)] calculated using the (measured) dynamic force change in response to TD ($\Delta F=m_{GAS}\cdot a_{COM}$) and either the displacement of the COM ($\Delta L_{MTC}$) or the fibre material elongation, respectively, both likewise measured in response to TD, $k_{TAC}$ was

12 $$\begin{aligned} k_{TAC}= & {} \frac{\Delta F\cdot k_{CE}}{\Delta L_{MTC}\cdot k_{CE}-\Delta F} \end{aligned}$$

there.

Here, however, the model is instead treated as a 3DoF symmetrical spring-mass system with at both ends fixated, i.e. a clamped (and pre-strained) system, where the distal-proximal symmetry in both stiffness [Eqs. (2) and  (3)] and mass [Eq. (7)], makes the displacement of $x_C$ (Equations of motion, Supplementary Eq. S4, p. 2)

13 $$\begin{aligned} k_{SC2}\cdot x_C-k_{SC}\cdot x_{S,p}-k_{SC}\cdot x_{S,d}= & {} \frac{1}{3}\cdot m_{GAS}\cdot a_{COM}=\frac{1}{3}\cdot \Delta F \end{aligned}$$

14 $$\begin{aligned} k_{SC2}\cdot x_C-k_{SC}\cdot \frac{x_{C}}{2}-k_{SC}\cdot \frac{x_{C}}{2}= & {} \frac{1}{3}\cdot \Delta F \end{aligned}$$

15 $$\begin{aligned} x_C= & {} \frac{\frac{1}{3}\cdot \Delta F}{(\frac{1}{k_S}+\frac{1}{k_{C}})^{-1}}, \end{aligned}$$

assuming that the displacement $m_{S,p}$ is half of $m_C$. When further assuming that $\Delta L_{MTC}=x_C$ is the same as in Eq. (12), being a measured quantity for the centre of mass displacement after TD, then

16 $$\begin{aligned} k_{S}=\frac{ k_{C}\cdot \Delta F}{3\cdot \Delta l_{MTC} \cdot k_{C}-\Delta F} , \end{aligned}$$

which makes the ratio between $k_{TAC}$ [Eq. (12)] inferred from experiments^11^ and our model parameter $k_S$ [Eq. (16)] correspond to

17 $$\begin{aligned} k_{TAC}\approx c_i\cdot k_{S}, \end{aligned}$$

where we find $c_a=3.7$ and $c_p=2.7$ for active and passive GAS, respectively (Supplementary Text S3). Likewise, the model parameter to determine fibre material stiffness is

18 $$\begin{aligned} k_{C}=\frac{k_{S}\cdot k_{SSC}-k_{S}^2}{2\cdot k_S-k_{SSC}}= 2\cdot k_{CE}, \end{aligned}$$

because the length of the CE region (which spans the GAS’ COM) at the instant before TD ($L_{CE,0}$) is a quantity used to estimate Young’s modulus of the fibre material $E_{CE}$ = 1.3 MPa^11^. To keep Young’s modulus the same, the stiffness of the fibre material must be ${k_{C}}=k_{CE}\cdot 2$ at $\frac{L_{CE}}{2}$, which is the length of one $k_C$ spring:

19 $$\begin{aligned} E_{CE}= \frac{k_{CE}\cdot L_{CE}}{ACSA_{avr}} =\frac{k_C\cdot L_{CE}}{2\cdot ACSA_{avr}} . \end{aligned}$$

## Statistical analysis

All the statistical tests were calculated using a one-way ANOVA with the Bonferroni adjustment for multiple comparisons, with the values presented as mean ± SD. Differences were statistically significant when *p* $\,\le \,\alpha$ = 0.05 or *p* $\,\le \,\alpha ^*$ = $\alpha$/n for the Bonferroni correction, with *n* being the number of performed tests.

## Results

In our spectral analysis, we determined eight frequencies, which we labelled $F1-F8$.

**Table 2:** Frequencies in active and passive trials.

Force ($\bar{F}_{max,p}$)	P1 (n = 6)	P2 (n = 7)	P3 (n = 6)	P4 (n = 7)
	0.21 N	0.14 N	0.16 N	0.13 N
Trial	1 cm	1.5 cm	4 cm	alu (2 mm$^\dagger$)
*F*1 (Hz)	56 ±2.0	56 ± 3.7	58 ± 5.7	–
*F*2 (Hz)	**131 ± 12.1**	**135 ± 3.4**	**139 ± 5.5**	**141 ± 5.2**
*F*3 (Hz)	**210 ± 14.6**	**208 ± 10.3**	**214 ± 8.7**	**211 ± 15.1**
*F*4 (Hz)	**281 ± 10.5**	**277 ± 10.2**	**287 ± 13.1**	**287 ± 13.2**
*F*5 (Hz)	338 ± 5.0	–	351 ± 10.6	357 ± 13.6
*F*6 (Hz)	–	–	–	–
*F*7 (Hz)	454 ± 14.8	449 ± 19.7	–	458 ± 14.9
*F*8 (Hz)	552 ± 17.2	–	545 ± 7.1	549 ± 3.7

Force ($\bar{F}_{max}$)	A1 (n = 6)	A2 (n = 8)	A3 (n = 7)	A4 (n = 6)
	22.6 N	25.9 N	24.6 N	27.8 N
Trial	1 cm	1.5 cm	4 cm	Alu (2 mm$^\dagger$)
*F*1 (Hz)	57 ± 8.6	66 ± 6.6	61 ± 8.7	–
*F*2 (Hz)	**165 ± 19. 6**	**164 ± 11.0**	**161 ± 4.3**	**168 ± 9.3**
*F*3 (Hz)	**263 ± 12.9**	**277 ± 16.8**	**263 ± 16.1**	**265 ± 19.7**
*F*4 (Hz)	–	–	–	–
*F*5 (Hz)	–	–	330 ± 19.6	336 ± 13.1
*F*6 (Hz)	**392 ± 19.1**	**400 ± 13.9**	**409 ± 12.1**	**400 ± 18.3**
*F*7 (Hz)	467 ± 17.3	466 ± 2.6	489 ± 20.2	465 ± 19.9
*F*8 (Hz)	–	–	550 ± 12.1	547 ± 7.6

The table lists the most dominant frequencies in all passive (P1, P2, P3, P4) and active (A1, A2, A3, A4) trials. P1$\vert$A1, P2$\vert$A2, P3$\vert$A3 and P4$\vert$A4 are subdivisions of trials in group1 (1 cm), group2 (1.5 cm), group3 (4 cm) and group4 (aluminium, 2 mm), respectively. The force underneath each label is either the mean passive force ($\bar{F}_{max,p}$) or the mean isometrically, active force ($\bar{F}_{max}$) measured by the force transducer just before TD in each group, respectively. Frequencies marked in bold are considered GAS frequencies.

$^{\dagger }$ The impact force measured by the force transducer in the aluminium trials (P4$\vert$A4) was closest to the impact force measured in the 1.5 cm trials (P2$\vert$A2).

**Figure 2:**
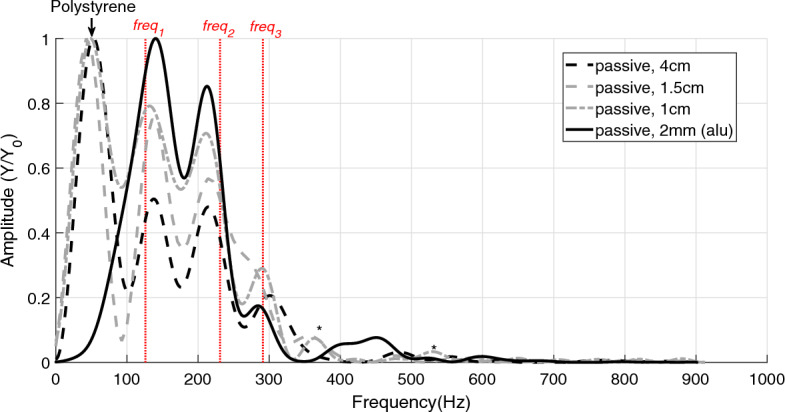
Frequencies in passive GAS when dropping the frame onto two different ground materials. The dashed, black and dashed, grey lines show the frequencies in GAS when the frame drops from either 4 cm or 1.5 cm onto polystyrene, respectively. The dash-dotted, grey line shows the frequencies in GAS when dropping the frame from 1 cm onto polystyrene. The black, solid line shows the frequencies in GAS when dropping the frame from 2 mm onto aluminium. The impact strength, when the frame drops onto aluminium, corresponds to a frame drop from 1.5 cm onto polystyrene, see Table 4. The red, vertical, dotted lines are the three model-predicted frequencies ($freq _{1}$, $freq _{2}$, and $freq _{3}$) of passive (P1) GAS [Eqs. (9) and (10)]. $^{*}$ A frequency that is also found in the FFT analysis of the frame (Table 4).

**Figure 3:**
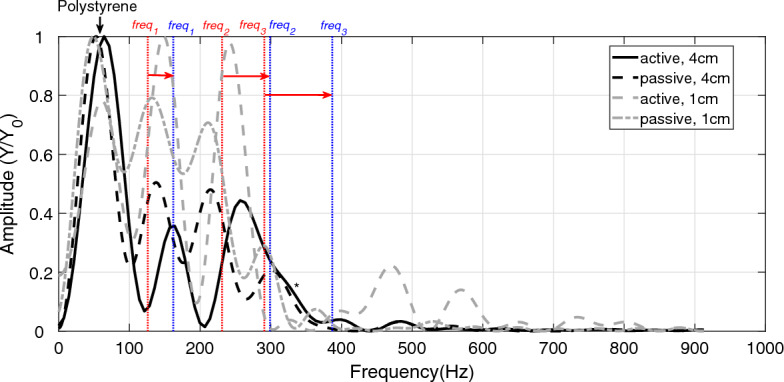
Examples of frequencies in passive and active muscle trials. The black line show the frequencies in active GAS when dropping the frame from 4 cm onto polystyrene. The dashed, black (4 cm) and dashed, grey (1 cm) lines are the frequencies of passive or active GAS, respectively, dropped onto polystyrene. The dash-dotted, grey line shows the frequencies in GAS when dropping the frame from 1 cm onto polystyrene. The red and blue, vertical, dashed lines, and their red arrows, indicate the shifts in model-predicted GAS frequencies from passive (P1, red) to active (A1, blue) GAS in group 1. *A frequency that is also found in the FFT analysis of the frame (Table 4).

When comparing these measured frequencies in more detail in active and passive GAS, we found no significant differences between the frequencies labelled *F*1, *F*5, and *F*8, whereas *F*2 and *F*3 significantly differed and can thus be attributed to GAS (compare Tables 2 and 3). Frequencies *F*1, *F*5, and *F*8 can be attributed to eigenfrequencies of the frame (compare Tables 2 and 4) interacting with the ground material or oscillating in itself. *F*7 significantly differ when comparing passive and active GAS (p = 0.02, Table 5) but is close to similar eigenfrequencies of the frame interacting with the ground material. The other four frequencies (*F*2, *F*3, and $F4$ $\vert$ $F6$, Table 5) measured in the passive and active GAS conditions can hence be concluded to reflect GAS eigenfrequencies. Further, *F*4 (see both top and bottom in Table 2, and compare with Table 3, top, $freq _{3}$) was not present in the active trials, whereas, in the passive trials, *F*6 (see both top and bottom in Table 2, and compare with Table 3, bottom, $freq _{3}$) was not present (Table 2, top and Fig. 2). The difference in active and passive GAS frequencies is, as an exemplary case, visualised in Fig. 3.

**Table 3:** Literature data used to estimate GAS eigenfrequencies. Both active (A) and passive (P) values for $k_{CE}$ and $k_{TAC}$ were taken from literature^11^ for scaling them to the spring stiffness values of $k_S$ [Eq. (17)] and $k_{C}$ [Eq. (18)], which were then used to estimate GAS eigenfrequencies (P1 and A1, respectively): $freq _{1,3}$ [Eq. (9)] and $freq _{2}$ [Eq. (10)].

$$k_{CE}$$$$^{{\textbf {*}}}$$	$$k_{TAC}$$$$^{{\textbf {*}}}$$	$$k_{C}$$	$$k_S$$	$$freq _{1}$$	$$freq _{2}$$	$$freq _{3}$$
Passive (P)						
3200 $\frac{\textrm{N}}{\textrm{m}}$	1844 $\frac{\textrm{N}}{\textrm{m}}$	6400 $\frac{\textrm{N}}{\textrm{m}}$	683 $\frac{\textrm{N}}{\textrm{m}}$	126 Hz	229 Hz	293 Hz
Active (A)						
15,000 $\frac{\textrm{N}}{\textrm{m}}$	4150 $\frac{\textrm{N}}{\textrm{m}}$	30,000 $\frac{\textrm{N}}{\textrm{m}}$	1121 $\frac{\textrm{N}}{\textrm{m}}$	162 Hz	298 Hz	386 Hz

*$k_{CE}$ and $k_{TAC}$ with $L_{CE}$ = 7.5 mm^11^ for both the passive and active trials (See Supplementary Fig. S1)

**Table 4:** Eigenfrequencies of the frame excluding GAS.

	*F*1	*F*2	*F*3	*F*4	*F*5
Frame (FFT, poly)	61 Hz ± 5.6	234 Hz ± 8.3	337 Hz ± 16.7	444 Hz ± 16.6	556 Hz ± 2.8
Frame (FFT, alu)	96 Hz ± 5.5	246 Hz ± 4.3	355 Hz ± 11.2	452 Hz ± 13.9	545 Hz ± 8.9

Both frame (FFT, poly) and frame (FFT, alu) list the eigenfrequencies of the frame, determined with either polystyrene or aluminium as ground material, respectively.

The replacement of polystyrene by aluminium as the ground material generally removed *F*1 at about 60 Hz from the measured spectra (*P*4 and *A*4, Table 2). Still, the remaining frequencies neither changed significantly with ground material in the active nor in the passive trials, except *F*2, slightly, within the passive trials (135 Hz and 141 Hz, p = 0.043, see Table 2).

**Figure 4:**
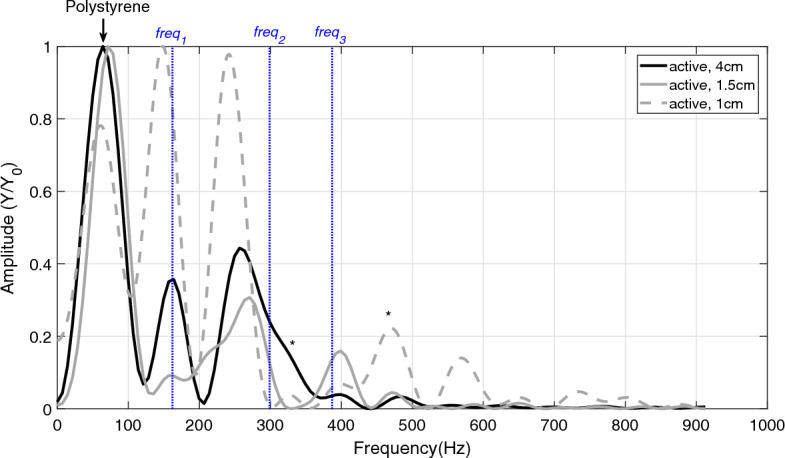
Frequencies in active muscle for three different drop heights. The black line indicates the GAS frequencies when dropping the frame from 4 cm onto polystyrene. The grey line shows the GAS frequencies when the frame drops from 1.5 cm, and the dashed, grey line shows the GAS frequencies when dropping the frame from 1 cm onto polystyrene. The blue, vertical, and dotted lines are the three model-predicted frequencies ($freq _{1}$, $freq _{2}$, and $freq _{3}$) of active (A1) GAS [Eqs. (9) and (10)]. *A frequency that is also found in the FFT analysis of the frame (Table 4).

Increasing the impact strength by dropping height (1 cm, 1.5 cm and 4 cm trials) did not cause significant changes in passive GAS frequencies. This likewise applies to the active trials (Fig. 4), with the only exception being the (slight) difference between A2 (466 Hz) and A3 (489 Hz, p = 0.035 in Table 2). Despite the frame eigenfrequencies *F*5 and *F*8 being present in some of the passive 1.5 cm trials, and *F*7 in the 4 cm trials, these were excluded from Table 2, because they were not present in every trial. Likewise, *F*5 in A1 and A2 are not included in Table 2. For statistical comparisons of active and passive trials, see Supplementary Table S1.

**Table 5:** Mean frequencies in active and passive trials.

	P1, P2, P3, P4	A1, A2, A3, A4
	Passive (n = 26)	Active (n = 27)
*F*1 (Hz)$^{\dagger }$	58 ± 5.8	61 ± 8.2
*F*2 (Hz)	**139 ± 11.8**	163 ± 12.0*
*F*3 (Hz)	**215 ± 13.3**	265 ± 18.2*
*F*4 (Hz)	**286 ± 12.1**	–
*F*5 (Hz)$^{\dagger }$	343.1 ± 14.7	334 ± 13.8
*F*6 (Hz)	–	**399 ± 17.4**
*F*7 (Hz)	455 ± 13.4	470 ± 14.6*
*F*8 (Hz)$^{\dagger }$	551 ± 12.8	552 ± 10.5

The middle column lists the mean values of the frequencies in all passive trials (P1, P2, P3, P4), whereas the right column lists the mean values of all the frequencies in the active trials (A1, A2, A3, A4). The frequencies are labelled *F*1–*F*8, starting from the lowest frequency found, and frequencies marked in bold are considered GAS frequencies. See Table 2 for frequencies measured in all passive and active GAS trials.

*The frequencies found were significantly different in passive and active GAS.

$^{\dagger }$ Assumed to be a frame frequency, see Table 4

As seen in Eqs. (9) and (10), only the two composite stiffnesses $k_{SSC}$ and $k_{SC2}$ are used to determine the eigenfrequencies of the model. If the stiffness values of active GAS (Table 3, centre, bottom) are used as an input to the model, then the 3DoF model-predicted eigenfrequencies are 162 Hz, 298 Hz, and 386 Hz (Table 3, bottom, right). The deviations of these theoretical eigenfrequencies relative to the average values of the measured active GAS frequencies (*F*2, *F*3, and *F*6 in Table 2, A1) are − 1.8% ($\frac{(162-165)\,\text {Hz}}{165\,\text {Hz}}$), 13.3% ($\frac{(298-263)\,\text {Hz}}{263\,\text {Hz}}$), and − 1.5% ($\frac{(386-392)\,\text {Hz}}{392\,\text {Hz}}$), respectively. In the passive GAS, the 3DoF model-predicted eigenfrequencies are 126 Hz, 229 Hz, and 293 Hz (Table 3, top, right). These deviate by -3.8% ($\frac{(126-131)\,\text {Hz}}{131\,\text {Hz}}$), 9.0% ($\frac{(229-210)\,\text {Hz}}{210\,\text {Hz}}$), and 4.3% ($\frac{(293-281)\,\text {Hz}}{281\,\text {Hz}}$), respectively, from their measured counterparts (*F*2, *F*3, and *F*4 in Table 2, P1).

## Discussion

When comparing the frequencies in Tables 2 and 4, it is likely that the frequencies at about 60 Hz, 330 Hz, 355 Hz, 450 Hz and 550 Hz reflect properties of the interaction between the frame itself and the ground material because these frequencies are still present without the GAS fixated in the frame. Further, the frequency at $\approx$ 60 Hz disappears in the FFT analysis when changing the ground material from polystyrene to aluminium. Thus, the $\approx$ 60 Hz in the FFT analysis is attributed to the polystyrene-frame interaction.

Across all trials, the frequencies attributed to GAS are always lower in passive trials (Table 5). As all three GAS frequencies (*F*2, *F*3, and $F4$ $\vert$ $F6$) depend on (active and passive) fibre material properties (Table 3: through the basic model stiffnesses $k_{C}$ and $k_{S}$, Fig. 1c, thus $k_{SC}$ and $k_{SSC}$, see Eqs. (3) and (4)], the positive correlation between frequency and muscle activity is likely due to forming cross-bridges. In active fibre material, attaching myosin heads mechanically couple actin and myosin filaments by building cross-bridges, and the filament sliding requires their distortion^31,32^. Furthermore, any added cross-bridge increases the fibre material stiffness^31,33^. In a study^11^ similar to our present one, we found that the stiffness in fully activated fibre material only (not $k_{S}$ representing fibre-aponeurosis properties, but $k_{C}$ here equalling $k_{CE}$ there^11^) is $\approx$ 370% ($\frac{15,000\,\text {N m}^{-1}-3200\,\text {N m}^{-1}}{3200\,\text {N \, m}^{-1}}$) higher than in the passive fibre material^11^ (see also Supplementary Table S4). Active muscle contraction also biaxially loads the aponeurosis^27,34^, and at least one study^21^ has shown in isotonic contraction experiments that the longitudinal stiffness of the probed aponeuroses of wild turkeys’ *M. gastrocnemius lateralis* increased by $\approx$ 64% ($\frac{180\,\text {N \, m}^{-1}-110\,\text {N \, m}^{-1}}{110\,\text {N \, m}^{-1}}$, [Fig. 5B^21^]) from the passive to the fully activated muscle condition. We assume that either the aponeuroses^17,21^ or fibre material^11^ dominate *F*2 due to their estimated stiffness values being at least an order of magnitude lower than that of the longer (proximal) GAS tendon, with tendon stiffness estimates being based on published values of Young’s modulus^30,31^. Unfortunately, with our present 3DoF model and current experimental setup, we can still not resolve the individual tissue contributions or potential differences in proximal and distal stiffness values.

One frequency (*F*7) that we found in the passive (455 Hz) condition did significantly differ from its active counterpart (470 Hz). Both of these frequencies are also slightly different from any frame or frame-ground-material frequencies (polystyrene, Table 4), nor does the 3DoF model predict them. Therefore, either the 3DoF model is too simple (e.g., too few degrees of freedom) to predict these frequencies or that the interaction between GAS and both frame extrusions slightly alters the overall stiffness of the muscle preparation. If the latter is the case, then this might also affect *F*2 in Table 4, whereas the lowest frame-ground-material frequency found remains the same because the frame mass and polystyrene visco-elasticity determine it.

For the active and passive trials, changing the impact strength leads to no significant changes in the frequency spectrum. Our findings might differ with a higher impact strength because for a fresh and fully stimulated GAS, the impact strength in the 4 cm trials is insufficient to induce forcible cross-bridge disruption^17^. Here, the impact strength is not enough to interfere significantly with the work-stroke: whether by forcible rupture of cross-bridge bonds^35^ or influence of the centre of oscillation at which the cross-bridge generates GAS’ maximum contractile force. Changing the impact strength may alter the oscillation amplitudes, however, issues like potential elastic recoil by wobbling masses, their dissipating energy, and wave propagation, all as functions of impact strength and thus their oscillation amplitudes, have not been a part of this study, although very worth while investigating next.

There were no significant differences in active GAS frequencies when comparing the aluminium (2 mm) trials and the 1.5 cm polystyrene trials (Supplementary Table S2). A likely explanation and an advantage of our setup is the controlled environment in which we can design impact situations on GAS that are complicated or impossible to specifically aim at in situ. For example, the lower limb muscles are not pre-activated in a tuned fashion^1,12–14^ in our experiments, and the lower limb is not pre-angled^36,37^, respectively, which allows us to reduce the expected complexity of the vibrational soft tissue response (here, e.g., focussing on longitudinal oscillation modes solely). In our results, we then find that in the pretty low-dimensional solution space of just a few frequencies only one of them, namely, *F*2 in the passive 1.5 cm trials (135 Hz) differs significantly from the likewise passive aluminium trials (141 Hz), with about the same impact strength (Table 2). We suspect that this (slight albeit significant: p = 0.043, Supplementary Table S1) difference in *F*2 may be accounted for by either the time between the last active trial and the passive one performed, or the time between dissection and the passive experiments, or the total number of experiments for a specific muscle. Such suspected memory or history mechanism may be structure-inherent: An appropriate hypothesis would be that this originates from the third filament, the giant molecule titin. Titin likely contributes $\ge$ 75% of the passive stiffness in fibre material at $L_{opt}$^11^, and titin is a visco-elastic material^38,39^. Titin’s mechanical effects, e.g. through its interaction with actin^40^, are determined by the contractile (both mechanical and activity) history and, thus and accordingly, vary with the time between each stretch/shortening contraction^38,41^. Unfortunately, again, due to formal restrictions (so far, only a few animals are available in each experimental session), and the overall methodical complexity of the setup, our sample sizes are currently simply too small, and our data is too few, to explore any of these potential correlations, up to now.

In general, our model-predicted GAS muscle eigenfrequencies match well in both cases probed in experiments, the passive (*P*1) and the fully active (*A*1). We show that treating the muscle as a system clamped by an alternating sequence of pre-strained, linear springs and portions of muscle mass (i.e. a system of serially coupled harmonic oscillators) quite accurately estimates GAS eigenfrequencies in response to the impact at TD, when at least two criteria are met: First, the muscle must be, initially (at TD), in near-isometric conditions, which is in agreement with the literature, as the angle flexion in the knee and ankle joint within the first 20 ms after TD is just $\approx$ 3° for *Tupaia glis* trotting at 1 m s^−1^^42^; i.e. GAS is stiff at TD, and both joint angular velocities are close to zero. The second criterion probably allows for more scope in active muscle length before TD, as contracting fibre material can compensate for any initial slackness. We also show that the elasticities of both aponeuroses and the fibre material dominate the high amplitude GAS eigenfrequencies and that any elasticity of tendon material on GAS eigenfrequencies are likely non-existent because of the differences in stiffnesses and mass compared to other GAS tissues. As rat GAS (distal) tendon length is not extraordinarily short, this should likewise apply to any other vertebrate of rat size or smaller^18,43^ because Young’s modulus^18,19^ then completely determines stiffness. Due to pure mechanical (inertia and compliance) reasons, in bigger animals with lower eigenfrequencies^17^, tendons may well play a significant role in muscle wobbling mass eigenfrequencies where $k_{t,d}$ and even $k_{t,p}$, with $k_{t,p} \ne k_{t,d}$, should be considered at first instance when analysing the eigenfrequencies of muscles bigger than rat GAS.

Whereas the first ($freq _{1}$) and third ($freq _{3}$) model-estimated eigenfrequencies (Table 3) are in almost perfect agreement with the two comparable experimentally found eigenfrequencies (*F*2 and $F4$ $\vert$ $F6$, Table 2), the second estimated eigenfrequency ($freq _{2}$) does deviate more from measured *F*3 than its counterparts. As we assume proximal and distal symmetry in both stiffness and mass, the absolute displacements of $m_{S,p}$ and $m_{S,d}$ are always the same (see Fig. 1c and our 3DoF model’s eigenvectors in Supplementary Eqs. S20, S22,  S23, p. 5, respectively). Therefore, our present model cannot cover any potential non-homogeneous fibre strain distribution (wave propagation) across the centre part (the belly made of fibres: CE, Fig. 1c) bar for the case of the eigenvector of $freq _{2}$ where the displacement of $m_{S,p}$ and $m_{S,d}$ is in opposite directions (Supplementary Eq. S20, p. 5). Not allowing fibre strain in any in-phase eigenvector contradicts literature that found $\approx$ 0.3% fibre strain at $F_{max}$ in wobbling mass experiments similar to our group3 trials^17^. To meet some fibre strain, either proximal-distal mass asymmetry or asymmetry in proximal and distal stiffnesses must be considered in the model. Any breaking of the proximal-distal model symmetry may include the necessity to improve our model explanations to the measured eigenfrequencies and -modes; yet, this demands to enhance the model complexity by adding degrees of freedom. Increasing the model complexity by adding degrees of freedom would, in turn, allow for better distinguishing anatomically defined tissue regions within GAS (i.e. exactly distinguishing and representing tendons, as well as mixed aponeurosis-fibre and pure fibre regions, respectively) and more transparently examining, and thus understanding, their contribution to the wobbling mass dynamics.

## Conclusion

Based on our findings, we conclude that the differences between the harmonic frequencies of impact-induced strain oscillations of passively clamped and actively contracting isometric rat GAS muscles are dominantly due to the attachment of myosin heads to actin (cross-bridges). That means the more cross-bridges, the higher the stiffness and the higher the GAS eigenfrequencies. Changing the GAS stiffness affects all frequencies that are certainly attributable to the muscle only in our experiments. We found that neither changing the impact strength, nor the ground material, influenced active or passive GAS frequencies significantly. With only three degrees of freedom, our model can explain at least two of the GAS frequencies measured in both the passive and the active conditions. It will be exciting to see whether very moderate enhancements of our present model (e.g. adding just one additional degree of freedom) are attending enhanced explanatory power regarding the wobbling mass dynamics.

### Supplementary Information

Supplementary Information.

## Data Availability

The datasets used and/or analysed during the current study available from the corresponding author on reasonable request.
