# Supplementary material for: Muscle wobbling mass dynamics: eigenfrequency dependencies on activity, impact strength, and ground material
Source: Sci Rep. 2023 Nov 9;13:19575. doi: 10.1038/s41598-023-45821-w (PMC10638252; doi:10.1038/s41598-023-45821-w)
Supplement: Supplementary file 1 — Supplementary Information. [file 41598_2023_45821_MOESM1_ESM.pdf]

Supplementary Information for

Muscle wobbling mass dynamics: eigenfrequency dependencies on activity, impact strength, and ground material

Kasper B. Christensen, Michael Günther, Syn Schmitt and Tobias Siebert

This PDF file includes:
Text 1-3
Table 1-4
Figure 1
References

Supplementary Text S1

Eigenfrequencies (3dof - fixated proximally and distally)

Equations of motion:

$$m_{S,p} \cdot \ddot{x}_{S,p} + (k_S + k_{SC}) \cdot x_{S,p} - k_{SC} \cdot x_C = 0 \tag{S1}$$

$$m_C \cdot \ddot{x}_C + (k_{SC} + k_{SC}) \cdot x_C - k_{SC} \cdot x_{S,p} - k_{SC} \cdot x_{S,d} = 0 \tag{S2}$$

$$m_{S,d} \cdot \ddot{x}_{S,d} + (k_{SC} + k_S) \cdot x_{S,d} - k_{SC} \cdot x_C = 0 \quad . \tag{S3}$$

Equations of motion (matrix):

$$\begin{bmatrix} m_{S,p} & 0 & 0 \\ 0 & m_C & 0 \\ 0 & 0 & m_{S,d} \end{bmatrix} \cdot \begin{bmatrix} \ddot{x}_{S,p} \\ \ddot{x}_C \\ \ddot{x}_{S,d} \end{bmatrix} + \begin{bmatrix} k_S + k_{SC} & -k_{SC} & 0 \\ -k_{SC} & k_{SC} + k_{SC} & -k_{SC} \\ 0 & -k_{SC} & k_{SC} + k_S \end{bmatrix} \cdot \begin{bmatrix} x_{S,p} \\ x_C \\ x_{S,d} \end{bmatrix} = \begin{bmatrix} 0 \\ 0 \\ 0 \end{bmatrix} \quad . \tag{S4}$$

Characteristic equation:

$$\left([k] - \omega^2 \cdot [m]\right) \cdot \begin{bmatrix} x_{S,p} \\ x_C \\ x_{S,d} \end{bmatrix} = 0 \quad . \tag{S5}$$

Determinant:

$$det\left([k] - \omega^2 \cdot [m]\right) = 0 \quad . \tag{S6}$$

Deduction of eigenfequencies of 3DoF model, assuming outer mass symmetry $m_{S,p} = m_{S,d} = m_S$ yet inequality of central mass $m_C \neq m_S$; the same for the stiffnesses $k_C \neq k_S$; see Fig. 1 in manuscript

We start with introducing some symbols and expressions

$$\begin{aligned}
k_{SC} &= \frac{k_S \cdot k_C}{k_S + k_C} \quad , \\
k_{SC2} = 2 \cdot k_{SC} &= 2 \cdot \frac{k_S \cdot k_C}{k_S + k_C} \quad , \\
k_{SSC} = k_S + k_{SC} &= \frac{k_S \cdot (k_S + 2 \cdot k_C)}{k_S + k_C} = k_S \cdot \left(1 + \frac{k_C}{k_S + k_C}\right) \quad , \\
k_{SSC} - k_{SC2} = k_S - k_{SC} &= \frac{k_S^2}{k_S + k_C} \quad , \\
\frac{k_{SSC}}{k_{SC2}} &= \frac{k_S + 2 \cdot k_C}{2 \cdot k_C} \quad , \\
\frac{2 \cdot (k_{SSC} - k_{SC2})}{k_{SC2}} &= \frac{k_S}{k_C}
\end{aligned} \tag{S7}$$

that will be useful later.

The symbol ω represents an angular eigenfrequency of the clamped 3DoF model oscillating, and we abbreviate $\lambda = \omega^2$. The characteristic equation determining the angular eigenfrequencies of the 3DoF model, with outer mass and stiffness symmetry, but central parameters different from peripheral ones ($m_C \neq m_S$, $k_C \neq k_S$; physiologically: $k_C > k_S$), requires the determinant of the (three equations of motion coupled in one dimension) to vanish:

$$\begin{vmatrix} k_{SSC} - \lambda \cdot m_S & -k_{SC} & 0 \\ -k_{SC} & k_{SC2} - \lambda \cdot m_C & -k_{SC} \\ 0 & -k_{SC} & k_{SSC} - \lambda \cdot m_S \end{vmatrix} = 0 \quad . \tag{S8}$$

Applying the rule of Sarrus, this yields a cubic equation

$$\begin{aligned}
& (k_{SSC} - \lambda \cdot m_S) \cdot (k_{SC2} - \lambda \cdot m_C) \cdot (k_{SSC} - \lambda \cdot m_S) \\
& -(-k_{SC})^2 \cdot (k_{SSC} - \lambda \cdot m_S) \\
& -(k_{SSC} - \lambda \cdot m_S) \cdot (-k_{SC})^2 \\
= \; & 0 \\
\Leftrightarrow \quad & (k_{SSC} - \lambda \cdot m_S) \cdot \big((k_{SSC} - \lambda \cdot m_S) \cdot (k_{SC2} - \lambda \cdot m_C) - 2 \cdot k_{SC}^2\big) \\
= \; & 0 \quad ,
\end{aligned} \tag{S9}$$

in terms of λ, which writes as a the product of a term linear in λ and another quadratic one in λ.

The product to vanish is fulfilled by either

$$k_{SSC} - \lambda \cdot m_S = 0 \quad , \tag{S10}$$

which yields the square of one angular eigenfrequency

$$\lambda_2 = \omega_2^2 = \frac{k_{SSC}}{m_S} = \frac{k_S}{m_S} \cdot \left(1 + \frac{k_C}{k_S + k_C}\right) \tag{S11}$$

here eventually given in terms of the basic model parameters m_S, m_C, k_S, and k_C. The remaining two angular eigenfrequencies are determined by otherwise the second factor of

the left hand side of Eq. S9 vanishing, i.e. by solving the corresponding quadratic equation

$$\begin{aligned} & (k_{SSC} - \lambda \cdot m_S) \cdot (k_{SC2} - \lambda \cdot m_C) - 2 \cdot k_{SC}^2 \\ & = 0 \\ \Leftrightarrow \quad & m_S \cdot m_C \cdot \lambda^2 - (m_S \cdot k_{SC2} + m_C \cdot k_{SSC}) \cdot \lambda + (k_{SSC} \cdot k_{SC2} - 2 \cdot k_{SC}^2) \\ & = 0 \quad , \end{aligned} \tag{S12}$$

for λ, which eventually yields

$$\begin{aligned} \lambda_{1,3} &= \omega_{1,3}^2 \\ &= \frac{k_{SC2}}{2 \cdot m_C} \cdot \left(1 + \frac{m_C}{m_S} \cdot \frac{k_{SSC}}{k_{SC2}} \pm \sqrt{1 + \left(\frac{m_C}{m_S} \cdot \frac{k_{SSC}}{k_{SC2}}\right)^2 - 2 \cdot \frac{m_C}{m_S} \cdot \frac{(k_{SSC} - k_{SC2})}{k_{SC2}}}\right) \\ &= \frac{k_S \cdot k_C}{m_C \cdot (k_S + k_C)} \cdot \left(1 + \frac{m_C}{m_S} \cdot \left(1 + \frac{k_S}{2 \cdot k_C}\right) \pm \sqrt{1 + \left(\frac{m_C}{m_S} \cdot \left(1 + \frac{k_S}{2 \cdot k_C}\right)\right)^2 - \frac{m_C}{m_S} \cdot \frac{k_S}{k_C}}\right) \quad . \end{aligned} \tag{S13}$$

If full mass symmetry is assumed ($m_S = m_C = m$), the squares of the angular eigenfrequencies according to Eq. S11 and Eq. S13, respectively, write

$$\omega_2^2 = \frac{k_{SSC}}{m} = \frac{k_S}{m} \cdot \left(1 + \frac{k_C}{k_S + k_C}\right) \quad , \tag{S14}$$

$$\omega_{1,3}^2 = \frac{k_S \cdot k_C}{m \cdot (k_S + k_C)} \cdot \left(2 + \frac{k_S}{2 \cdot k_C} \pm \sqrt{2 + \left(\frac{k_S}{2 \cdot k_C}\right)^2}\right) \quad . \tag{S15}$$

Correspondingly, the eigenfrequencies are

$$freq_i = \frac{\sqrt{\omega_i^2}}{2 \cdot \pi} \qquad with \qquad i = 1, 2, 3 \quad . \tag{S16}$$

That only the k_{SSC} stiffness is needed to determine $freq_2$ (Eq. S16) is because of the distal-proximal symmetry (Eq. S8), which makes the corresponding eigenvector (V_2) of $x_{S,p}$, x_C, and $x_{S,d}$ (Eq. S5) for $freq_2$ for $\lambda_2 = \frac{k_{SSC}}{m_S}$ (Eq. S11) equal to:

$$\begin{bmatrix} k_{SSC} - \lambda_2 \cdot m_S & -k_{SC} & 0 \\ -k_{SC} & k_{SC2} - \lambda_2 \cdot m_C & -k_{SC} \\ 0 & -k_{SC} & k_{SSC} - \lambda_2 \cdot m_S \end{bmatrix} \cdot V_{2:\frac{k_{SSC}}{m_S}} = \begin{bmatrix} 0 \\ 0 \\ 0 \end{bmatrix} \tag{S17}$$

$$\begin{bmatrix} 0 & -k_{SC} & 0 \\ -k_{SC} & k_{SC} - k_S & -k_{SC} \\ 0 & -k_{SC} & 0 \end{bmatrix} \cdot \begin{bmatrix} x_{S,p} \\ x_C \\ x_{S,d} \end{bmatrix} = \begin{bmatrix} 0 \\ 0 \\ 0 \end{bmatrix} \tag{S18}$$

$$\begin{bmatrix} 0 & -k_{SC} & 0 \\ -k_{SC} & k_{SC} - k_S & -k_{SC} \\ 0 & -k_{SC} & 0 \end{bmatrix} \cdot \begin{bmatrix} -1 \\ 0 \\ 1 \end{bmatrix} = \begin{bmatrix} 0 \\ 0 \\ 0 \end{bmatrix} \quad , \tag{S19}$$

with

$$V_{2:\frac{k_{SSC}}{m_S}} = \begin{bmatrix} -1 \\ 0 \\ 1 \end{bmatrix} \quad . \tag{S20}$$

Similarly, the eigenvectors for $\lambda_{1,3}$ (Gaussian elimination);

$$\begin{aligned}
&\left[\begin{array}{ccc|c} k_{SSC}-\lambda_1\cdot m_S & -k_{SC} & 0 & 0 \\ -k_{SC} & k_{SC2}-\lambda_1\cdot m_C & -k_{SC} & 0 \\ 0 & -k_{SC} & k_{SSC}-\lambda_1\cdot m_S & 0 \end{array}\right] \begin{matrix} R1\div(k_{SSC}-\lambda_1\cdot m_S)\to R1 \\ \\ R3\div(k_{SSC}-\lambda_1\cdot m_S)\to R3 \end{matrix} \\
\Longleftrightarrow\ &\left[\begin{array}{ccc|c} 1 & \frac{-k_{SC}}{k_{SSC}-\lambda_1\cdot m_S} & 0 & 0 \\ -k_{SC} & k_{SC2}-\lambda_1\cdot m_C & -k_{SC} & 0 \\ 0 & \frac{-k_{SC}}{k_{SSC}-\lambda_1\cdot m_S} & 1 & 0 \end{array}\right] \begin{matrix} \\ R2-(-k_{SC})\cdot(\frac{R2-(-k_{SC})\cdot R3}{-k_{SC}})\to R2 \\ R3\cdot(k_{SSC}-\lambda_1\cdot m_S)\to R3 \end{matrix} \\
\Longleftrightarrow\ &\left[\begin{array}{ccc|c} 1 & \frac{-k_{SC}}{k_{SSC}-\lambda_1\cdot m_S} & 0 & 0 \\ 0 & \frac{k_{SC}^2}{k_{SSC}-\lambda_1\cdot m_S} & -k_{SC} & 0 \\ 0 & -k_{SC} & k_{SSC}-\lambda_1\cdot m_S & 0 \end{array}\right] R2\div(\frac{k_{SC}^2}{k_{SSC}-\lambda_1\cdot m_S})\to R2 \\
\Longleftrightarrow\ &\left[\begin{array}{ccc|c} 1 & \frac{-k_{SC}}{k_{SSC}-\lambda_1\cdot m_S} & 0 & 0 \\ 0 & 1 & \frac{-k_{SC}\cdot(k_{SSC}-\lambda_1\cdot m_S)}{k_{SC}^2} & 0 \\ 0 & -k_{SC} & k_{SSC}-\lambda_1\cdot m_S & 0 \end{array}\right] \begin{matrix} R1-(\frac{-k_{SC}}{k_{SSC}-\lambda_1\cdot m_S})\cdot R2\to R1 \\ \\ R3-(-k_{SC})\cdot R2\to R3 \end{matrix} \\
\Longleftrightarrow\ &\left[\begin{array}{ccc|c} 1 & 0 & -1 & 0 \\ 0 & 1 & \frac{k_{SSC}-\lambda_1\cdot m_S}{-k_{SC}} & 0 \\ 0 & 0 & 0 & 0 \end{array}\right]
\end{aligned} \tag{S21}$$

are

$$V_{1:\frac{k_{SSC}-\lambda_1\cdot m_S}{k_{SC}}} = \begin{bmatrix} 0.6978 \mid 0.6826 \\ 1 \mid 1 \\ 0.6978 \mid 0.6826 \end{bmatrix} \tag{S22}$$

and

$$V_{3:\frac{k_{SSC}-\lambda_3\cdot m_S}{k_{SC}}} = \begin{bmatrix} -0.7165 \mid -0.7325 \\ 1 \mid 1 \\ -0.7165 \mid -0.7325 \end{bmatrix} \quad , \tag{S23}$$

when using m_S=m_C=0.00063 kg (Eq. 7 in main text) and either passive or active values for k_S=683 $\frac{\text{N}}{\text{m}}$ | 1121 $\frac{\text{N}}{\text{m}}$, k_{SC}=6400 $\frac{\text{N}}{\text{m}}$ | 30000 $\frac{\text{N}}{\text{m}}$ (Table 3 in main text), and $\lambda_{1,3}$ (Eq. S13).

Supplementary Text S2

Estimating spring stiffnesses values from eigenfrequencies

If instead $\omega = \sqrt{\omega_1^2} = \mathit{freq}_1 \cdot 2 \cdot \pi$ is used to to estimate the local stiffness of the suspended

3DoF system, and not the other way around, then by rewriting Eq. S13 where $m_{S,p} = m_{S,p} = m_S \neq m_C$ (case1):

$$\begin{aligned} & 2 \cdot \omega^2 - \frac{k_{SSC}}{m_S} - \frac{k_{SC2}}{m_S} \\ = & - \sqrt{(\frac{k_{SSC}}{m_S} + \frac{k_{SC2}}{m_C})^2 - 4 \cdot (\frac{k_{SSC}}{m_S} \cdot \frac{k_{SC2}}{m_C} - \frac{k_{SC2}}{m_S} \cdot \frac{k_{SC2}}{m_C} \cdot \frac{1}{2})} \end{aligned} \tag{S24}$$

$$\begin{aligned} & - \frac{4 \cdot k_{SSC} \cdot \omega^2}{m_S} - \frac{4 \cdot \omega^2 \cdot k_{SC2}}{m_C} + 4 \cdot \omega^4 + \frac{k_{SSC}^2}{m_S^2} + \frac{2 \cdot k_{SSC} \cdot k_{SC2}}{m_S \cdot m_C} + \frac{k_{SC2}^2}{{m_C}^2} \\ = & \frac{k_{SSC}^2}{m_S^2} - \frac{2 \cdot k_{SSC} \cdot k_{SC2}}{m_S \cdot m_C} + \frac{2 \cdot k_{SC2}^2}{m_S \cdot m_C} + \frac{k_{SC2}^2}{m_C^2} \end{aligned} \tag{S25}$$

$$\begin{aligned} & 4 \cdot m_S^2 \cdot m_C^2 \cdot \omega^4 - 4 \cdot k_{SSC} \cdot m_S \cdot m_C^2 \cdot \omega^2 - 4 \cdot m_S^2 \cdot m_C \cdot k_{SC2} \cdot \omega^2 \\ & + k_{SSC}^2 \cdot m_C^2 + 4 \cdot k_{SSC} \cdot m_S \cdot m_C \cdot k_{SC2} - 2 \cdot m_S \cdot m_C \cdot k_{SC2}^2 \\ = & k_{SSC} \cdot m_C^2 \end{aligned} \tag{S26}$$

$$\begin{aligned} 0 = & -(2 \cdot m_S \cdot m_C) \cdot k_{SC2}^2 + (-4 \cdot m_S^2 \cdot m_C \cdot \omega^2 + 4 \cdot k_{SSC} \cdot m_S \cdot m_C) \\ & \cdot k_{SC2} + (4 \cdot m_S^2 \cdot m_C^2 \cdot \omega^4 - 4 \cdot k_{SSC} \cdot m_C^2 \cdot m_S \cdot \omega^2) \quad . \end{aligned} \tag{S27}$$

From this (Eq. S27), the inferred spring stiffness $k_{SC} = \frac{k_{SC2}}{2}$ (using the quadratic formula) is equal to:

$$k_{SC} = -\frac{b_1 - \sqrt{b_1^2 + 8 \cdot m_S \cdot m_C \cdot (4 \cdot m_S^2 \cdot m_C^2 \cdot \omega^4 - 4 \cdot k_{SSC} \cdot m_S \cdot m_C^2 \cdot \omega^2)}}{8 \cdot m_S \cdot m_C} \quad , \tag{S28}$$

where

$$b_1 = 4 \cdot m_S^2 \cdot m_C \cdot \omega^2 - 4 \cdot k_{SSC} \cdot m_S \cdot m_C \quad . \tag{S29}$$

Herein, the spring stiffness k_{SSC} for case1, using Eq. S16 (for ω_2^2), is

$$k_{SSC} = (2 \cdot \pi \cdot freq_2)^2 \cdot m_S \quad . \tag{S30}$$

In the simpler 3DoF model variation, where $m_{S,p} = m_{S,d} = m_C = \frac{m_{GAS}}{3} = m$ (case2), the inferred stiffnesses k_{SC} and k_{SSC}(Eq. S28 and Eq. S30, respectively) then are

$$k_{SC} = \frac{b_2 - \sqrt{b_2^2 + 8 \cdot m^2 \cdot (4 \cdot m^4 \cdot \omega^4 - 4 \cdot k_{SSC} \cdot m^3 \cdot \omega^2)}}{4 \cdot m^4} \quad , \tag{S31}$$

where

$$b_2 = 4 \cdot m^3 \cdot \omega^2 - 4 \cdot k_{SSC} \cdot m^2 \quad , \tag{S32}$$

and

$$k_{SSC} = (2 \cdot \pi \cdot \mathit{freq}_2)^2 \cdot m \qquad , \tag{S33}$$

respectively.

Supplementary Text S3

Stiffness comparisons to literature

In a previous work that examined the wobbling characteristics of a muscle [1,2], the stiffness of the tendon-aponeurosis-complex (k_{TAC}), was inferred assuming the muscle was a simple spring mass system that is fixated proximally and has a lumped mass attached distally:

$$k_{MTC} = \left(\frac{1}{k_{TAC}} + \frac{1}{k_{CE}} \right)^{-1} \quad . \tag{S34}$$

With both k_{MTC} and k_{CE} (Eq. S34) calculated using the dynamic force change in response to TD ($\Delta F = m_{GAS} \cdot a_{COM}$) and either the measured displacement of the COM (ΔL_{MTC}) or the fibre material elongation in response to TD, respectively, k_{TAC} is equal to

$$k_{TAC} = \frac{\Delta F \cdot k_{CE}}{\Delta L_{MTC} \cdot k_{CE} - \Delta F} \quad . \tag{S35}$$

Here, however, the GAS is instead treated as a 3DoF symmetrical spring-mass system with both ends of the springs fixated, and because of the distal-proximal symmetry in our 3DoF model (see 'GAS 3DoF model: spring stiffness‘ and 'GAS 3DoF model: mass‘ in main text), the displacement of x_C (Eq. S2) is

$$k_{SC2} \cdot x_C - k_{SC} \cdot x_{S,p} - k_{SC} \cdot x_{S,d} = \frac{1}{3} \cdot m_{GAS} \cdot a_{COM} = \frac{1}{3} \cdot \Delta F \tag{S36}$$

$$k_{SC2} \cdot x_C - k_{SC} \cdot \frac{x_C}{2} - k_{SC} \cdot \frac{x_C}{2} = \frac{1}{3} \cdot \Delta F \tag{S37}$$

$$x_C = \frac{\frac{1}{3} \cdot \Delta F}{(\frac{1}{k_S} + \frac{1}{k_C})^{-1}} \tag{S38}$$

assuming that, firstly, $m_{S,p} = m_C = m_{S,d}$ (Eq. 7 in main text), secondly, that the displacement of the first mass is half of the second mass, and, thirdly, that $\Delta L_{MTC} = x_C$ is the same as in Eq. S35, because it is a measured quantity for the centre of mass displacement after TD. Accordingly,

$$k_S = \frac{k_C \cdot \Delta F}{3 \cdot \Delta l_{MTC} \cdot k_C - \Delta F} \quad . \tag{S39}$$

By comparing Eq. S34 and Eq. S39, and remembering that L_{CE} (used to calculate k_{CE}) spans across GAS' COM, then

$$k_C = 2 \cdot k_{CE} \quad , \tag{S40}$$

as the fibre material stiffness at $\frac{L_{CE}}{2}$ (the length of one k_C spring) must be $2 \cdot k_C = k_{CE}$ to keep Young's modulus of the fibre material the same as in Eq. S35. Therefore, by knowing k_{CE} (Supplementary Table 4),

$$k_{TAC} = 4286\,\frac{\mathrm{N}}{\mathrm{m}} = \frac{15000\,\frac{\mathrm{N}}{\mathrm{m}} \cdot 0.2\,\mathrm{N}}{6 \cdot 10^{-5}\,\mathrm{m} \cdot 15000\,\frac{\mathrm{N}}{\mathrm{m}} - 0.2\,\mathrm{N}} \tag{S41}$$

(Eq. S35), and

$$k_S = 1154\,\frac{\mathrm{N}}{\mathrm{m}} = \frac{30000\,\frac{\mathrm{N}}{\mathrm{m}} \cdot 0.2\,\mathrm{N}}{3 \cdot 6 \cdot 10^{-5}\,\mathrm{m} \cdot 30000\,\frac{\mathrm{N}}{\mathrm{m}} - 0.2\,\mathrm{N}} \tag{S42}$$

(Eq. S39), then the difference between k_{TAC} and k_S at F_{max} is a factor of

$$3.7 = c_a = \frac{4286\,\mathrm{N\,m^{-1}}}{1154\,\mathrm{N\,m^{-1}}} \quad , \tag{S43}$$

or

$$2.7 = c_p = \frac{1455\,\mathrm{N\,m^{-1}}}{542\,\mathrm{N\,m^{-1}}} \quad , \tag{S44}$$

when using values (active | passive) from Supplementary Table 4.

	P1	P2	P3		A1	A2	A3
$\mathbf{P2}_{F1}$	0.592			$\mathbf{A2}_{F1}$	0.278		
$\mathbf{P3}_{F1}$	0.151	0.271		$\mathbf{A3}_{F1}$	0.369	0.832	
$\mathbf{P4}_{F1}$	–	—	—	$\mathbf{A4}_{F1}$	—	—	—
$\mathbf{P2}_{F2}$	0.573			$\mathbf{A2}_{F2}$	0.939		
$\mathbf{P3}_{F2}$	0.327	0.102		$\mathbf{A3}_{F2}$	0.689	0.601	
$\mathbf{P4}_{F2}$	0.274	0.043	0.753	$\mathbf{A4}_{F2}$	0.6431	0.406	0.500
$\mathbf{P2}_{F3}$	0.667			$\mathbf{A2}_{F3}$	0.393		
$\mathbf{P3}_{F3}$	0.512	0.165		$\mathbf{A3}_{F3}$	0.939	0.404	
$\mathbf{P4}_{F3}$	0.836	0.257	0.700	$\mathbf{A4}_{F3}$	0.838	0.327	0.888
$\mathbf{P2}_{F4}$	0.469			$\mathbf{A1}_{F4}$	—		
$\mathbf{P3}_{F4}$	0.590	0.202		$\mathbf{A2}_{F4}$	—	—	
$\mathbf{P4}_{F4}$	0.403	0.108	0.759	$\mathbf{A3}_{F4}$	—	—	—
$\mathbf{P2}_{F5}$	—			$\mathbf{A2}_{F5}$	—		
$\mathbf{P3}_{F5}$	0.540	—		$\mathbf{A3}_{F5}$	0.939	—	
$\mathbf{P4}_{F5}$	0.051	—	0.519	$\mathbf{A4}_{F5}$	0.993	—	0.519
$\mathbf{P2}_{F6}$	—			$\mathbf{A2}_{F6}$	0.451		
$\mathbf{P3}_{F6}$	—	—		$\mathbf{A3}_{F6}$	0.259	0.383	
$\mathbf{P4}_{F6}$	—	—	—	$\mathbf{A4}_{F6}$	0.465	0.767	0.686
$\mathbf{P2}_{F7}$	0.619			$\mathbf{A2}_{F7}$	0.927		
$\mathbf{P3}_{F7}$	—	—		$\mathbf{A3}_{F7}$	0.103	0.035	
$\mathbf{P4}_{F7}$	0.712	0.417	—	$\mathbf{A4}_{F7}$	0.966	0.923	0.163
$\mathbf{P2}_{F8}$	—			$\mathbf{A2}_{F8}$	—		
$\mathbf{P3}_{F8}$	0.627	—		$\mathbf{A3}_{F8}$	—	—	
$\mathbf{P4}_{F8}$	0.773	—	0.366	$\mathbf{A4}_{F8}$	—	—	0.405

Supplementary Table S 1 | Differences in frequencies in GAS between groups (p-values).

	$F1$	$F2$	$F3$	$F4$	$F5$	$F6$	$F7$	$F8$
Active	—	0.406	0.506	—	—	0.569	0.986	0.554
Passive	—	0.043	0.485	0.108	—	—	0.417	—

Supplementary Table S 2 | GAS dropped onto two different ground materials with the same impact strength. p-values of trials dropped onto polystyrene (group2) and aluminium (group4).

	$F1$	$F2$	$F3$	$F4$	$F5$	$F6$	$F7$	$F8$
p-value	0.056	1e-12	1e-17	—	1e-4	—	0.011	0.625

Supplementary Table S 3 | Differences in frequencies in GAS for active and passive trials. The table compares (p-values) frequencies found in active (A1, A2, A3, A4) and passive trials (P1, P2, P3, P4). See also Table 2 in main text.

Description	Symbol	Data	Unit	Source [2]
Dynamic force change	ΔF	0.2	N	text
COM displacement after TD (23 N)	ΔL_{MTC}	$6 \cdot 10^{-5}$	m	$\frac{\Delta F}{k_{MTC}}$
MTC stiffness (23 N)	k_{MTC}	3250	$\mathrm{N\,m^{-1}}$	[Fig. S4]
Fibre material stiffness (23 N)	k_{CE}	15000	$\mathrm{N\,m^{-1}}$	[Fig. S4]
Tendon-aponeurosis-fibre stiffness (23 N)	k_{TAC}	4150	$\mathrm{N\,m^{-1}}$	$(\frac{1}{k_{MTC}} - \frac{1}{k_{CE}})^{-1}$
COM displacement after TD (p)	$\Delta L_{MTC,p}$	$2 \cdot 10^{-4}$	m	$\frac{\Delta F}{k_{MTC,p}}$
MTC stiffness (p)	$k_{MTC,p}$	1170	$\mathrm{N\,m^{-1}}$	[Fig. S4]
Fibre material stiffness (p)	$k_{CE,p}$	3200	$\mathrm{N\,m^{-1}}$	[Fig. S4]
Tendon-aponeurosis-fibre stiffness (p)	$k_{TAC,p}$	1844	$\mathrm{N\,m^{-1}}$	$(\frac{1}{k_{MTC,p}} - \frac{1}{k_{CE,p}})^{-1}$

Supplementary Table S4 | GAS Length, Force and stiffness values in response to impact. The data in the table are taken from the literature when GAS is either F_{max}=23 N or passive (p) and assumes that the length of the fibre region is $L_{CE,0}$=7.5 mm [2].The stiffness values in this table are also plotted in Supplementary Fig. S1.

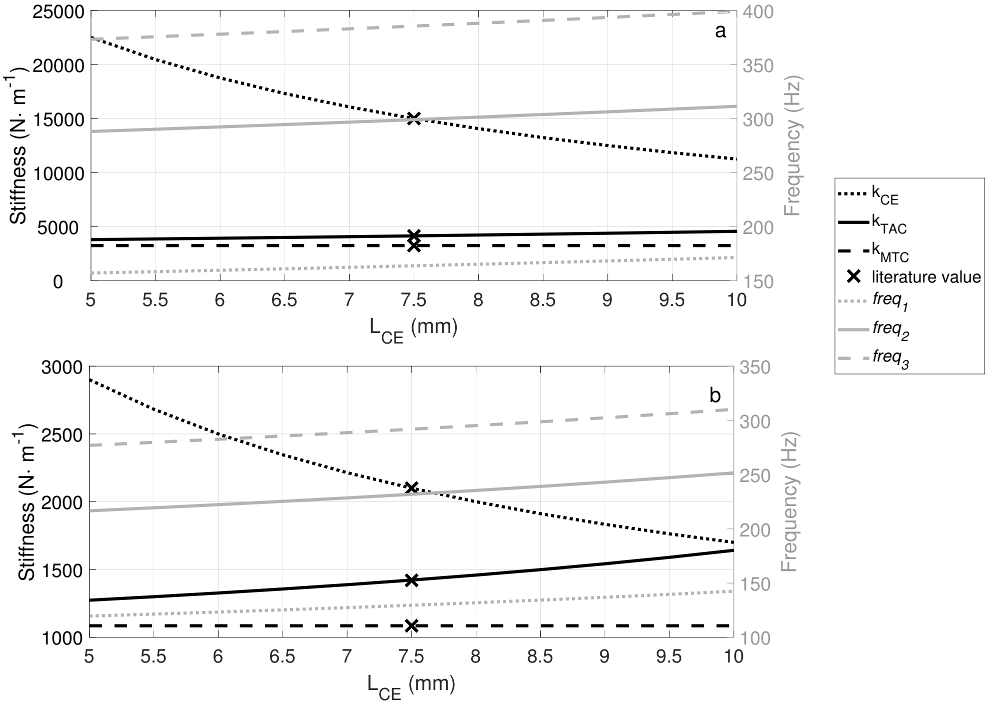

Supplementary Fig. S1 |GAS stiffnesses and eigenfrequencies as a function of the fibre length region. Depending on the lines they touch, the "X" symbols are the k_{CE}, k_{TAC} and k_{MTC} in either active (**a**) or passive (**b**) GAS. Here, the fibre material stiffness k_{CE} in both passive and active muscle (dotted, black lines) scales linearly with the change in fibre region length ($k_{CE} \cdot \frac{L_{CE}}{L_{CE,0}}$). As a result thereof, the k_{TAC} (Supplementary Eq. S34) changes (dashed black lines), whereas k_{MTC} remains constant (dashed, black lines). The right y-axis gives the 3DoF estimated eigenfrequencies in GAS: $freq_1$ (dotted, grey lines), $freq_2$ (solid, grey lines), and $freq_3$ (dashed, grey lines) illustrate the dependencies of the eigenfrequencies on the experimentally determined stiffness values (Supplementary Eq. S16) due to the lumped, regional stiffnesses k_{CE} and k_{TAC} (in accordance with the 1DoF model used for extracting these values from our experiments [1,2]) changing with the fibre-material-only length L_{CE} in various GAS specimens, while assuming full mass symmetry $m = \frac{m_{GAS}}{3}$ and a given value of Young's modulus of CE, 1.3 MPa [2]. The "X" values are from Supplementary Table S4.

References

1. Christensen, K. B., Günther, M., Schmitt, S. & Siebert, T. Strain in shock-loaded skeletal muscle and the time scale of muscular wobbling mass dynamics. Scientific Reports **7**, 13266 (2017).

2. Christensen, K. B., Günther, M., Schmitt, S. & Siebert, T. Cross-bridge mechanics estimated from skeletal muscles' work-loop responses to impacts in legged locomotion. Scientific Reports **11**, 23638 (2021).
